# Extra-Virgin Olive Oil Modifies the Changes Induced in Non-Nervous Organs and Tissues by Experimental Autoimmune Encephalomyelitis Models

**DOI:** 10.3390/nu11102448

**Published:** 2019-10-14

**Authors:** Cristina Conde, Begoña M. Escribano, Evelio Luque, Montserrat Feijóo, Javier Caballero-Villarraso, Manuel E. Valdelvira, Juan J. Ochoa-Sepúlveda, Rafael Lillo, Elier Paz, Abel Santamaría, Eduardo Agüera, Isaac Túnez

**Affiliations:** 1 Instituto Maimonides de Investigación Biomédica de Córdoba (IMIBC), Av. Menendez Pidal, 14004 Cordoba, Spain; cristinaconde84@gmail.com (C.C.); cm1lucae@uco.es (E.L.); monfey@hotmail.com (M.F.); bc2cavij@uco.es (J.C.-V.); mevaldelviradiaz@gmail.com (M.E.V.); ochoasepulveda@gmail.com (J.J.O.-S.); md1liror@uco.es (R.L.); doctoredu@gmail.com (E.A.); 2 Servicio de Neurología, Hospital Universitario Reina Sofía, 14004 Cordoba, Spain; 3 Departamento de Biología Celular, Fisiología e Inmunología, Facultad de Veterinaria, Universidad de Córdoba Campus of Rabanales, 14071 Cordoba, Spain; 4 Departamento de Ciencias Morfológicas, Sección de Histología, Facultad de Medicina y Enfermería, Universidad de Córdoba, 14004 Cordoba, Spain; 5 Departamento de Especialidades Médico-Quirúrgicas, Facultad de Medicina y Enfermería, Universidad de Córdoba, 14004 Cordoba, Spain; 6 Servicio de Análisis Clínicos, Hospital Universitario Reina Sofía, 14004 Cordoba, Spain; 7 Departamento de Bioquímica y Biología Molecular, Facultad de Medicina y Enfermería, Universidad de Córdoba, Av. Menendez Pidal, 14004 Cordoba, Spain; 8 Departamento de Ciencias Socio-sanitarias u Radiología y Medicina Física, Sección de Psiquiatría, Facultad de Medicina y Enfermería, Universidad de Córdoba, 14004 Cordoba, Spain; 9 Canvax Biotech S.L., 14014 Cordoba, Spain; e.paz@canvaxbiotech.com; 10 Laboratorio de Aminoácidos Excitadores, Instituto Nacional de Neurología y Neurocirugía, Mexico City 14269, Mexico; absada@yahoo.com; 11 Cooperative Research Thematic Excellent Network on Brain Stimulation (REDESTIM), Ministery for Economy, Industry and Competitiveness, 28046 Madrid, Spain

**Keywords:** extra-virgin olive oil, bacterial lipopolysaccharide, glutathione redox system, multiple sclerosis, extra-nervous tissues

## Abstract

This study reveals the existence of oxidative stress (reactive oxygen species (ROS)) in non-nervous organs and tissues in multiple sclerosis (MS) by means of a model of experimental autoimmune encephalomyelitis (EAE) in rats. This model reproduces a similar situation to MS, as well as its relationship with intestinal microbiota starting from the changes in bacterial lipopolysaccharide levels (LPS) in the outer wall of the gram-negative bacteria. Finally, the administration of extra-virgin olive oil (EVOO), hydroxytirosol (HT), and oleic acid (OA) exert beneficial effects. Twenty-five Dark Agouti two-month-old male rats, weighing around 190 g, were distributed into the following groups: Control, EAE (experimental autoimmune encephalomyelitis group), EAE + EVOO, EAE + HT, and EAE + OA. The glutathione redox system with the EAE was measured in heart, kidney, liver, and small and large intestines. The LPS and the correlation with oxidative stress in the small and large intestines were also investigated. The results showed that (1) the oxidative damage in the EAE model affects non-nervous organs and tissues; (2) The LPS is related to inflammatory phenomena and oxidative stress in the intestinal tissue and in other organs; (3) The administration of EVOO, HT, and OA reduces the LPS levels at the same time as minimizing the oxidative damage; (4) EVOO, HT, and OA improve the disease’s clinical score; and (5) on balance, EVOO offers a better neuroprotective effect.

## 1. Introduction

Multiple sclerosis (MS) is a chronic, inflammatory and neurodegenerative disease, which affects the central nervous system (CNS) [1,2,3,4]. The cause is unknown, but it appears to involve a combination of genetic susceptibility [5] and nongenetic triggering, such as a virus, metabolism, or environmental factors that together result in a self-sustaining autoimmune disorder, leading to recurrent immune attacks on the CNS [4,6,7,8,9].

The immune cell access to the CNS causes an increase of pro-inflammatory molecules from lymphocytes, inducing the production of reactive oxygen species (ROS) and a depletion of antioxidant systems [10,11,12], causing damage to mitochondria and myelin, oligodendrocyte apoptosis, and astrocyte dysfunction, characteristics of MS [13,14]. Increases in oxidative stress markers were observed in the cerebrospinal fluid, blood, and plasma of patients with relapsing-remitting MS (RR-MS) [14,15,16,17,18]. This background could suggest the idea of ROS and their inflammatory presence in organs outside of the CNS during the development of MS, which could lead to a vicious circle.

Different studies have indicated the role of gut microbiota dysfunctions in the development of experimental autoimmune encephalomyelitis (EAE) in rat and mouse, such as in the experimental model of MS [19,20,21,22,23,24,25]. In addition, the evidence shows a relationship between the neuroendocrine system and microbiota [26] that might not only affect the CNS but also other organs. It has been proposed that bacterial lipopolysaccharides (LPS) can cause the activation of the immune system and hypothalamic–pituitary–adrenal (HPA) axis [26].

A large body of evidence supports the beneficial effects of the Mediterranean diet in preventing neurodegeneration. Within the Mediterranean, diet olive oil is one of its principal and common components. The beneficial effects on health of extra-virgin olive oil (EVOO) have also been attributed to its mono-unsaturated fats content and to the presence of phenolic compounds that have antioxidant, anti-inflammatory, and immunomodulatory properties [27,28,29,30]. However, only Liuzzi et al. [31] studied the effect of an olive oil extract on the concentration of matrix metalloproteinase (MMP) 9 (MMP-9; gelatinase B) and MMP-2 (gelatinase A; matrix metallopeptidase 2) in rat astrocytes stimulated with LPS and in serum samples from MS patients, suggesting that olive oil might be useful in inhibiting MMP activity implicated in the course of the inflammatory responses observed in MS. In fact, MMP-9 increases the permeability of the blood brain barrier (BBB), facilitating the infiltration of leukocytes into the CNS, and causing myelin degradation as well as neuronal damage during the course of MS [32,33,34]. Moreover, two works analyzed the protective action of oleanic acid and erythrodiol, triterpenes present in olive oil [32,35,36]. Conde et al. [25] recently verified the beneficial effects of EVOO and two of its components, hydroxytirosol (HT) and oleic acid (OA), on the improvement of oxidative stress in the CNS and blood of EAE rats, reducing lipid peroxidation (LPO) product levels and carbonylated proteins, and boosting the role of glutathione peroxidase (GPx) in brain, spinal cord, and blood. They also related those favorable effects of olive oil to the intestinal microbiota starting from the diminution in LPS levels and its carrier protein (lipopolysaccharide binding protein (LBP)) in the three tissues cited above [25]. However, not yet analyzed are the likely effects of EAE on other extra-nervous organs and tissues and the possible repercussion of EVOO on the course of the disease.

In view of these antecedents, the main objective of this study was to establish the fact that the EAE model, which reproduces a similar clinical picture to RR-MS, triggers effects in extra-nervous organs and tissues, and our secondary objectives were (1) to analyze the effects of EVOO and two of its components, HT and OA, on the organs studied during the course of the EAE; (2) to verify if the LPS, a reflection of the intestinal microbiota status, is correlated with EAE oxidative stress and whether it could be responsible for the oxidative damage in organs different from the CNS, and (3) to prove if EVOO, HT, and OA modify the microbial LPS levels.

## 2. Material and Methods

### 2.1. Animals

On the basis of the guidelines of the Directive of 24 November 1986 (86/609/ECC) approved by the European Communities Council and RD 53/2013 (BOE, 8 February, 2013), the Bioethics Committee approved the protocols (number project 07/11/2018/157).

A total of 25 Dark Agouti male rats (2 months old, weighing around 190 g) from the Animal Experimentation Center (Córdoba University) were used. All of them were housed under standard colony conditions: 12:12 light/darkness cycle (lights on at 07:00), controlled room temperature (22 ± 2 °C), with free access to food and water.

### 2.2. Experimental Protocol

On the basis of previous studies [25], this study included the following study groups (*n* = 5 animals per group): control group (not manipulated); EAE group; EAE + extra-virgin olive oil (EVOO); EAE + hydroxytirosol group (HT); and EAE + oleic acid group (OA). 

EAE induction was performed by injecting subcutaneously, at the dorsal base of the tail, 100 μL of a solution containing 150 μg of myelin oligodendrocyte glycoprotein (MOG) (fragment 35–55; Sigma–Aldrich, Madrid, Spain) in phosphate buffered saline (PBS) emulsified 1:1 in complete Freund’s adjuvant (Sigma–Aldrich, St. Louis, Missouri, MO, USA) completed with 400 μg of heat-inactivated Mycobacterium tuberculosis (H37Ra, DIFCO, Detroit, MI, USA); dietary treatments began 14 days after injection.

The EVOO used in the present work was “Los Montes de Luque” gifted by Almazara de Luque S.C.A. (Olivarera Nuestra Señora del Rosario^®^, D.O.P. Baena; Luque, Cordoba, Spain). This EVOO (900 kcal/3389 KJ for a bottle of 5 L) is an own-label oil of Albenzaide EVOO D.O.P (Almazara de Luque S.C.A. at the above address). HT was purchased from Seprox Biotech S.L. (Madrid, Spain) and OA from Merck (Darmstadt, Germany). In the group given EVOO, the latter represented 10% of their calorie intake (in terms of weight) [25] in the total standard daily diet of rats AIN-93G [37], while OA (the acidity of the EVOO was of 0.17% OA/0.5 L EVOO) corresponded to 4% of the calorie intake. Both EVOO and OA were dietary substitutes. The HT group received 2.5 mg/kg body weight [25]. EVOO, OA, and HT were administered for 51 days with a gastric catheter. The administration started after 14 days, in other words, after the induction of EAE and the first evaluation of the scores.

### 2.3. Sample Preparation and Study

At 65 days, the animals were sacrificed by decapitation, having been previously anesthetized with an intraperitoneal injection of Ketamine 75 mg/kg (Imalgene^®^ 100 mg/mL, Merial Laboratorios).

Then, and under controlled temperature conditions, the heart, liver, kidney, and small and large intestines were extracted and weighed to immediately prepare the corresponding homogenates with a mechanical homogenizer (Tempest Virtis). The samples were homogenized in Tris (20 mM) at pH 7.4.

The analyses were performed in duplicate. Oxidative damage biomarkers in heart, liver, kidney, and small and large intestines were all analyzed by spectrophotometry with Bioxytech S.A. reagents (Oxis International; Portland, OR, USA) and with a Shimadzu spectrophotometer (UV 1603; Kyoto, Japan). The monitored biomarkers included lipid peroxidation products (LPO; nmol/mg protein) and those from the glutathione redox system: total glutathione (tG; nmol/mg protein), reduced glutathione (GSH; nmol/mg protein) and oxidized glutathione (GSSG; nmol/mg protein). For the determination of the glutathione peroxidase (GPx; nmol/mg protein), the Flohé and Gunzler [38] method was used. Carbonylated proteins (CP) were measured (nmol/g protein) using the Levine et al. [39] method. The GSH/GSSG ratio was also found.

LPS was assessed using the 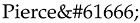 data are expressed as endotoxin units/mg protein. LBP assessment was performed using the Elisa Kit soluble LBP (Enzo^®^, Enzo Life Sciences (ELS), NY, USA). The amount of LBP was measured in an Elisa reader. The values are presented as pg/mg protein.

### 2.4. Clinical Score Evaluation

The animals were monitored at 14 and 65 days and scored in accordance with the following severity scale: (0) no signs, (1) tail paralysis, (2) weakness in hind legs, (3) paralysis in hind legs, (4) paralysis in hind legs and weakness in front legs, and (5) quadriplegic [25,40]. The difference (increase) between the score at 65 days and the score at 14 days was established**.**

### 2.5. Statistics

The statistical study was performed with the SPSS application (SPSS INC. Version 15 for Windows, Armonk, NY, USA). The data were expressed as mean ± SD. All groups showed a normal distribution so that a one-way ANOVA corrected with Bonferroni’s post-hoc test was used. The Pearson correlation coefficient was applied to assess the relationship between LPS and LBP with LPO and CP in the small and large intestines. The level of statistical significance was set at *p* < 0.05.

## 3. Results

### 3.1. Oxidative Stress in Different Body Organs

In the heart, with EAE, tG and GPx increased (*p* < 0.001) while in the kidney, the GSH and GSSG diminished (*p* < 0.001) with respect to the control (Table 1). In EAE, CP (*p* < 0.001) and LPO (*p* < 0.001) significantly increased in the heart, kidney, and liver (Table 2).

**Figure 1:**
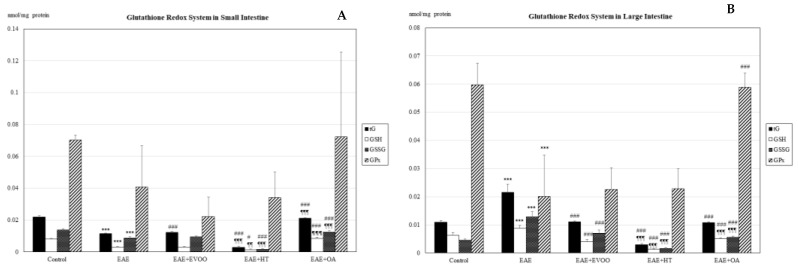
Glutathione redox system in the small intestine (**A**) and large intestine (**B**): total glutathione (tG; nmol/mg protein), reduced glutathione (GSH; nmol/mg protein), oxidized glutathione (GSSG; nmol/mg protein), and glutathione peroxidase (GPx; nmol/mg protein) in the following study groups: control group (not manipulated); EAE group (experimental autoimmune encephalomyelitis induced by myelin oligodendrocyte glycoprotein (MOG)); EAE + extra-virgin olive oil (EVOO); EAE + hydroxytirosol group (HT); and EAE + oleic acid group (OA). *** *p* < 0.001 vs. control; # < *p* < 0.05 vs. EAE; ### *p* < 0.001 vs. EAE; ¶¶ *p* < 0.01 vs. EAE + EVOO; ¶¶¶ *p* < 0.001 vs. EAE + EVOO.

**Figure 2:**
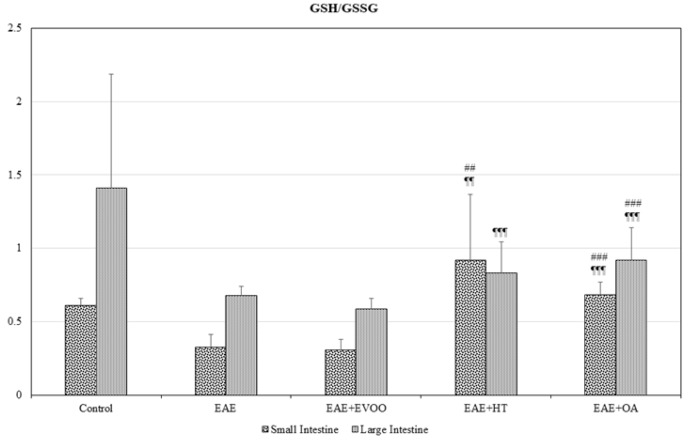
GSH/GSSG ratio in small and large intestine: reduced glutathione/oxidized glutathione ratio in the following study groups: control group (not manipulated); EAE group (experimental autoimmune encephalomyelitis induced by myelin oligodendrocyte glycoprotein (MOG)); EAE + extra-virgin olive oil (EVOO); EAE + hydroxytirosol group (HT); and EAE + oleic acid group (OA). ## < *p* < 0.01 vs. EAE; ### *p* < 0.001 vs. EAE; ¶¶ *p* < 0.01 vs. EAE + EVOO; ¶¶¶ *p* < 0.001 vs. EAE + EVOO.

**Figure 3:**
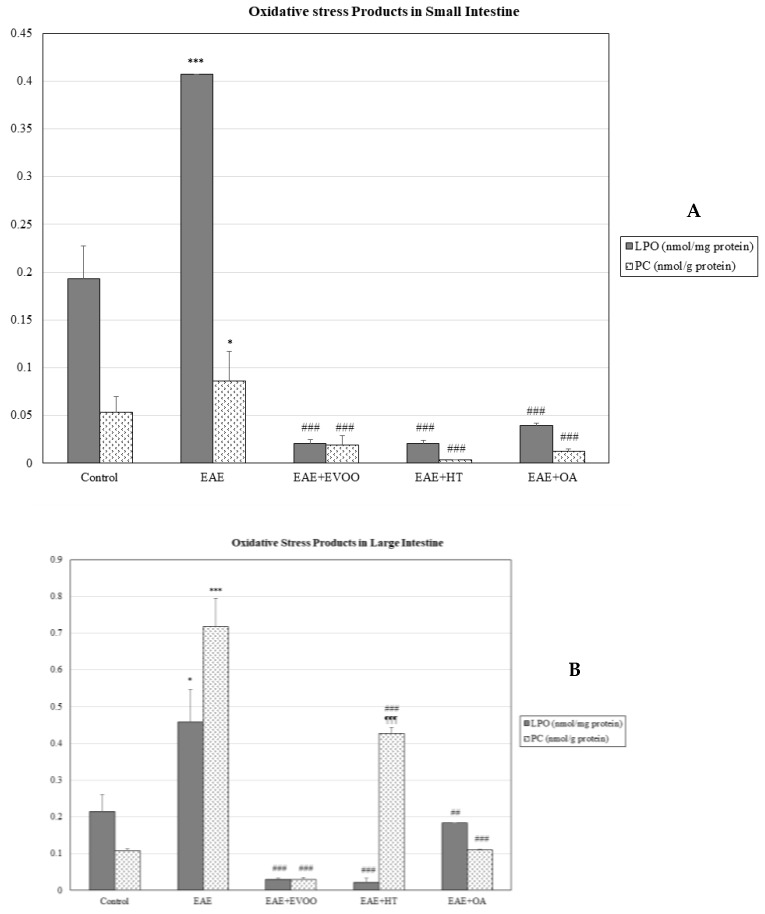
Oxidative stress products in the small intestine (**A**) and large intestine (**B**): lipid peroxidation products (LPO; nmol/mg protein) and carbonylated proteins (CP; nmol/g protein) in the following study groups: control group (not manipulated); EAE group (experimental autoimmune encephalomyelitis induced by myelin oligodendrocyte glycoprotein (MOG)); EAE + extra-virgin olive oil (EVOO); EAE + hydroxytirosol group (HT); and EAE + oleic acid group (OA). * *p* < 0.05 vs. control; *** *p* < 0.001 vs. control; ## < *p* < 0.01 vs. EAE; ### *p* < 0.001 vs. EAE; ¶¶¶ *p* < 0.001 vs. EAE + EVOO.

In the small intestine, EAE produced a significant decline (*p* < 0.001) in the tG, GSH, and GSSG (Figure 1A), whereas in the large intestine, on the contrary, there was a significant increase (*p* < 0.001) in them, together with a significant decrease in the GPx (*p* < 0.001) (Figure 1B). In both intestines, the GSH/GSSG ratio significantly decreased (Figure 2) and LPO and CP were significantly increased (Figure 3A,B).

### 3.2. EVOO, HT, and OA against the Oxidative Stress of EAE

In the small intestine, the EVOO produced a significant increase in tG (*p* < 0.001) with respect to the EAE, whereas OA significantly augmented not only tG (*p* < 0.001), but also GSH (*p* < 0.001) and GSSG (*p* < 0.001) with respect to EAE. Conversely, HT significantly diminished tG (*p* < 0.001), and so did GSH (*p* < 0.05) and GSSG (*p* < 0.001) with respect to the EAE values (Figure 1A). The GSH/GSSG ratio significantly increased with HT and OA with respect to EAE (Figure 2). The mean values obtained for tG, GSH, GSSG, and the GSH/GSSG ratio with HT and OA are seen to be significantly different from those obtained with EVOO (Figure 1A and Figure 2).

In the large intestine (Figure 1B), EVOO, HT, and OA significantly reduced the tG, GSH, and GSSG values with respect to EAE rats. Only OA produced significant increases in the GPx (*p* < 0.001) and GSH/GSSG ratio (Figure 2) with respect to EAE. The mean values for GSH (*p* < 0.0019), GSSG (*p* < 0.001), and the GSH/GSSG ratio (*p* < 0.001) with HT and OA are seen to be different from those observed with EVOO, and also tG (*p* < 0.001) for HT in comparison with EVOO (Figure 1B and Figure 2).

In the heart, tG and GPx underwent a significant decrease (*p* < 0.001) with EVOO and HT with regard to EAE animals. OA caused a significant increase in tG (*p* < 0.001) and a reduction in GPx (*p* < 0.001) with respect to EAE animals. The tG and GPx for HT and OA were significantly different (*p* < 0.001) from the mean values obtained with EVOO (Table 1).

In the kidney, only OA significantly increased GSSG (*p* < 0.001) with respect to EAE, whereas EVOO and HT significantly reduced its values (*p* < 0.001). With regard to GSH, EVOO and OA significantly increased (*p* < 0.001) its values with respect to EAE, and HT (*p* < 0.001) reduced them. In general, the mean GSH and GSSG values with HT and OA were significantly different (*p* < 0.001) from those observed with EVOO (Table 1).

The GSH/GSSG ratio was increased with EVOO in the heart, kidney, and liver with respect to EAE (*p* < 0.001), whereas the ratio values with HT and OA were seen to be significantly diminished with respect to EVOO (*p* < 0.001) and did not significantly differ from those obtained with EAE, except in the heart for OA (*p* < 0.001) (Table 1).

LPO (*p* < 0.001) and CP (*p* < 0.001) values were significantly reduced with EVOO, HT, and OA in all the organs studied (heart, kidney, liver, and small and large intestines) (Table 2 and Figure 3A,B).

### 3.3. Correlation of LPS and LBP with LPO and CP

**Figure 4:**
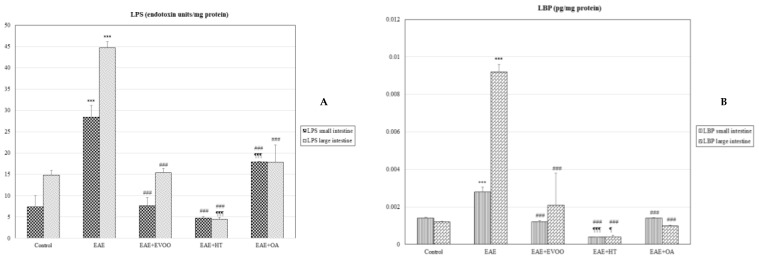
LPS (endotoxin units/mg protein (**A**) and LBP (pg/mg protein (**B**) in the small and large intestines: lipopolysaccharide (LPS) of the outside wall of the gram-negative bacteria and lipopolysaccharide binding protein (LBP) in the following study groups: control group (not manipulated); EAE group (experimental autoimmune encephalomyelitis induced by myelin oligodendrocyte glycoprotein (MOG)); EAE + extra-virgin olive oil (EVOO); EAE + hydroxytirosol group (HT); and EAE + oleic acid group (OA). *** *p* < 0.001 vs. control; ### *p* < 0.001 vs. EAE; ¶ *p* < 0.05 vs. EAE + EVOO; ¶¶¶ *p* < 0.001 vs. EAE + EVOO.

LPS and LBP were positively correlated with LPO and CP in both the small and large intestines (Table 3). Furthermore, LPS and LBP were significantly increased with EAE (Figure 4A,B).

### 3.4. EVOO, HT, and OA against the Microbiota

LPS (*p* < 0.001) and LBP (*p* < 0.001) were significantly reduced with EVOO, HT, and OA with respect to the EAE group. The mean values obtained with HT for LBP were lower than those observed with EVOO in the small (*p* < 0.001) and large intestines (*p* < 0.05) (Figure 4A,B).

### 3.5. Clinical Score at 65 Days minus 14 Days and Correlation with LPS, LBP, LPO, and CP

**Figure 5:**
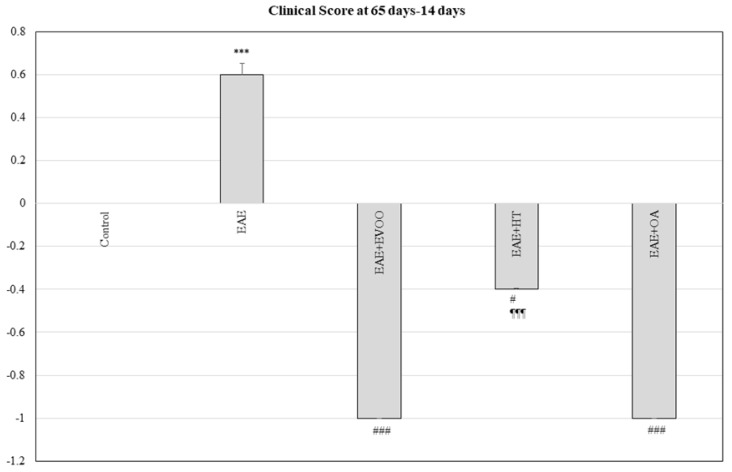
Clinical Score at 65 days less 14 days: The animals were monitored at 14 and 65 days and scored in accordance with the following severity scale: (0) no signs, (1) tail paralysis, (2) weakness in hind legs, (3) paralysis in hind legs, (4) paralysis in hind legs and weakness in front legs, and (5) quadriplegic [23,40]. The increase between the score at 65 days less the score at 14 days was established for the following study groups: control group (not manipulated); EAE group (experimental autoimmune encephalomyelitis induced by myelin oligodendrocyte glycoprotein (MOG)); EAE + extra-virgin olive oil (EVOO); EAE + hydroxytirosol group (HT); and EAE + oleic acid group (OA). *** *p* < 0.001 vs. control; # *p* < 0.05 vs. EAE; ### *p* < 0.001 vs. EAE; ¶¶¶ *p* < 0.001 vs. EAE + EVOO.

EVOO (*p* < 0.001), HT (*p* < 0.05), and OA (*p* < 0.001) produced a decrease in the clinical score at 65 days compared to the score given at 14 days, the opposite to what happened in the EAE group in which there was an increase (*p* < 0.001) in the clinical score with respect to the control. This diminution was greater with EVOO and OA, the effects produced by the HT being significantly different from those caused by EVOO (*p* < 0.001) (Figure 5).

There was a positive correlation between LPS, LBP, LPO, and CP with the clinical score at 65 days less 14 days (*p* < 0.05) in both the small and large intestines.

## 4. Discussion

This study shows, and confirms for the first time as far as we know, the presence of an intense oxidative stress in non-nervous organs in the model of EAE, enabling us to infer that something similar could occur in patients suffering from MS. This fact has been indirectly endorsed by previous studies on blood in the EAE model, showing that the changes in oxidative stress and inflammation biomarkers are similar to those found in the blood of patients with RR-MS [23]. In addition, and interestingly, this work, also for the first time, proves the protective effect both of EVOO, and of its compounds on non-nervous organs (heart, kidney, liver, and small and large intestines) affected by oxidative damage in the EAE model.

Up to now, oxidative stress produced during MS and EAE has been evidenced in the following target organs: brain, spinal cord, blood, serum, and cerebrospinal fluid [14,15,16,17,18,23]. However, it has not been elucidated whether this oxidative stress could be present in other extra-nervous organs during the disease.

Different expression patterns have recently been revealed in the rat CNS of the collectin surfactant protein-A (SP-A) in inflammatory response modulation in EAE. Also, in vitro treatment of human astrocytes and microglia with LPS promoted SP-A expression in a dose-dependent manner [41]. In fact, its levels were lower in the cerebrospinal fluid of patients with MS [42,43]. SP-A significantly decreases Toll-like receptor 4 and nuclear factor-κB expression, and reduces interleukin-1β and tumor necrosis factor-α levels [41].

The protein SP-A is not only present in the CNS but also in extra-nervous tissues. Thus, it has been localized in the lung, where it plays a basic role in pulmonary homeostasis and inflammatory response, and also in several extrapulmonary tissues among which the intestine, colon, and mucosa are included [41,44,45]. That discovery led us to develop the idea that the inflammation and oxidative stress accompanying MS and EAE could also be present in other extra-nervous tissues where the protein SP-A could express itself.

This actually happened in our results. The LPO and CP concentrations, revelatory of oxidative damage, increased in the heart, kidney, liver and large and small intestines as a result of the EAE. The glutathione redox system, one of the principal organic, antioxidant systems (and the main intracellular one), was also seen to be altered with respect to the individual control, in all the organs studied in EAE rats. That corroborated that the oxidative stress was produced in these extra-nervous organs due to both the production of ROS and to the alteration in body antioxidant defenses.

Accumulating evidence indicates that regular consumption of EVOO, the main source of fat in the Mediterranean diet, is associated with a reduced risk of developing chronic, degenerative disorders such as cardiovascular diseases, type 2 diabetes, and cancer [30]. EVOO is obtained from olives solely by mechanical or other physical preparation methods, under conditions that do not alter its natural composition [46]. EVOO is characterized by very high contents both of monounsaturated fatty acids (mainly oleic acid) and antioxidant molecules (mainly phenolic compounds) [47]. Hydroxytyrosol (3,4-dihydroxyphenylethanol) and tyrosol (3-hydroxyphenylethanol) are considered to be the most abundant and representative phenolic alcohols in olive oil [48,49,50].

Thus, olive oil administered to rats subjected to brain hypoxia–reoxygenation was demonstrated to exert antioxidant and cytoprotective activity, decreasing brain cell death, LPO level overproduction, counteracting the decrease in glutathione levels, and inhibiting prostaglandin E2 (PGE2) in brain tissues [32,51]. Likewise, long-term, polyphenols-rich extra-virgin olive oil dietary administration in mice counteracted age-related dysfunctions in motor coordination and increased GPx activity in some brain regions such as the cortex and cerebellum [32,52]. Nevertheless, there are few studies on the effects of EVOO administration on the oxidative stress caused by EAE and MS. A recent one from our team [25] disclosed the neuroprotective and antioxidant power of EVOO, HT, and OA on the brain, spinal cord, and blood against oxidative stress produced by EAE. However, up to now, this antioxidant capacity has not been demonstrated in other body organs also affected by EAE, although it is known that EVOO can be widely distributed over the organic territory. Hydroxytyrosol 14C radioactivity measured in different tissues showed that EVOO spreads to skeletal muscles, liver, heart, kidney, lung, and brain [32,53].

In our study, EVOO returned to normal the changes induced by EAE in GSH, GSSG, the GSH/GSSG ratio, tG, and GPx in the heart, the kidney, and the small and large intestines. A similar effect was noted with the administration of HT and OA.

Also, EVOO, HT, and OA showed the same effectiveness in the reduction of the oxidative metabolism products CP and LPO in all the organs studied.

Although it will be necessary to study the complex molecular mechanisms through which, specifically, EVOO, HT, and OA act on the oxidative stress produced by the EAE, it has been shown that there is a positive correlation between CP and LPO with LPS and LBP in the small and large intestines, and of all of them with the clinical score of 65 days less 14 days.

In a recent publication, our group demonstrated that the LPS and LBP levels in the brain, spinal cord, and blood of rats with EAE, and in the blood of patients with RR-MS, were high, together with other parameters representative of oxidative stress. Treatments with natalizumab, N-acetyl cysteine (NAC), and dimethyl fumarate (DMF) reduced LPS and LBP levels and the oxidative damage present in EAE and MS [23]. The incorporation into the diet of EVOO, HT, and OA also achieved similar results in the three body structures studied [25]. LPS and LBP had a positive correlation with all the parameters, revealing oxidative stress [23,25]. At least part of the oxidative effects noted in the nervous and blood tissues was associated with the changes triggered in LPS and LBP, a sign of possible modifications in the gut microbiota. These facts have been backed up in previous data from our group, as well as in scientific literature data showing the relationship between the gut microbiota and the CNS in what is known as the gut microbiota–brain axis.

One possible explanation for this gut microbiota–brain axis is in a work by Buscarine et al. [22], who discovered that changes in intestinal permeability (IP) are presented in RR-MS, with a possible genetic influence on the determinants of IP changes (as has been inferred from data on twins); IP changes included a deficit of the active mechanism of absorption from the intestinal lumen. These data support the idea that the gut may play an important role in the development of MS. Findings suggest that the relationship between the gut-microbiota composition and host blood immune markers can differ between children with and without MS [54], which supports the hypothesis of this IP in MS.

In our study, LPS and LBP appear to be increased in EAE and positively correlated with the oxidative stress present in the small and large intestines, which are affected by the disease. That This study showed that endotoxins in the intestinal microbiota could travel not only to the CNS through the IP, demonstrated during MS [22], but also to other body organs, and is responsible for inflammatory phenomena and the resulting oxidative damage typical of the EAE. Upregulation of ICAM-1 and VCAM-1 and the changes in the expression of these molecules in the brain blood vessels by the LPS have already been demonstrated [55,56]. These endothelial alterations would be responsible for the diapedesis of the immune cells and for the cerebral inflammatory phenomena causing the oxidative damage during the EAE and MS. Also, oxidative stress stimulates the adhesion of monocytes to the vascular endothelium and it modifies the permeability of the blood–brain barrier so that an intense peripheral stress in RR-MS patients has been observed [57]. Therefore, this inflammatory phenomenon could be repeated in the same way in each of the organs in this study affected by the EAE. In fact, there are signs that activation of the HPA axis by the gut microbiota can occur as a result of increased permeability of the intestinal barrier and a microbiota-driven pro-inflammatory state [26,58].

Finally, EVOO, HT, and OA significantly reduced the LPS and LBP values in intestinal tissue, showing their effectiveness in the fight against bacterial endotoxins, although future studies will be necessary to determine the action mechanism of this process, and its repercussions on that of the EAE in the heart and kidney, where EVOO, HT, and OA have exhibited their efficacy in overcoming oxidative stress and in contributing to the clinical improvement of the disease

It could be thought that, as in the brain, the bacterial endotoxins from the dysbiosis of the intestinal microbiota travels via the bloodstream not only to the brain vessels but also to other extra-nervous tissues, where it is responsible for the EAE inflammatory phenomenon and oxidative stress due to its alteration in the vascular permeability. EVOO, HT, and OA not only improve the inflammatory phenomenon for its patent activity on the gelatinases (A and B), with a role evidenced in the alteration of blood permeability, but they act on the oxidative stress, sequestering the free radicals that are inhibitors of cyclooxygenase, Fe^2+^, and NO, besides improving motor coordination, as has been demonstrated in Alzheimer [32].

The weaknesses of this study could lie in it not having demonstrated that the molecular action mechanisms witnessed in nervous tissue during the EAE and MS also occur in other extra-nervous tissues or that EVOO, HT, and OA act on the bacterial endotoxin itself and the inflammatory phenomena and oxidative stress triggered by the latter. However, this paper is of indisputable value in that it reveals that there is oxidative stress in non-nervous organs during the EAE, including in the intestinal tissue, in which an increase in bacterial endotoxin was observed; the paper also shows the correlation existing between the LPS levels and the oxidative stress-derived products (LPO and CP) in the intestine and the substantial improvement that the EVOO, HT, and OA exercised on the oxidative and clinical stress in the disease, as well as its role in reducing bacterial endotoxin levels.

## 5. Conclusions

Therefore, based on all that was expounded above, the conclusions of this study are:

(1) The oxidative damage of the EAE not only affects the CNS but also the principal body organs (small and large intestines, liver, kidney, and heart).
(2) The bacterial microbiota endotoxin seems to be implicated in the production of inflammatory phenomena and subsequent oxidative stress in the intestinal tissue and in other organs.
(3) Treatment with EVOO, HT, and OA reduces the bacterial endotoxin levels in the intestines at the same time as minimizing the oxidative damage in extra-nervous organs.
(4) EVOO, HT, and OA improve the clinical score of the disease itself.

Taken together, these data highlight the excellent effects of EVOO and its neuroprotective potential. They show that the incorporation and regularity of EVOO in the diet (as a nutritional intervention) would improve the general inflammatory status and, thus, the nerve and peripheric involvement of the patients, underlining its possible use as an adjuvant agent in MS treatments. Future studies will be necessary for:

(1) verifying the mechanism by which the intestinal microbiota is responsible for the inflammatory phenomena and the oxidative damage produced by the EAE and MS, not only in the CNS, but also in other organs;
(2) finding out the molecular action mechanisms of LPS in the phenomena leading to the transendothelial migration of the immune system in the CNS and other organs affected by the EAE and MS; and
(3) identifying the action mechanism/s of the EVOO in its protective effects in EAE and MS.

## Figures and Tables

**Table 1:** Glutathione redox system in the heart, kidney, and liver.

	Glutathione Redox System				
	**Heart**				
	tG (nmol/mg protein)	GSH (nmol/mg protein)	GSSG (nmol/mg protein)	GSH/GSSG	GPx (nmol/mg protein)
Control	0.01086 ± 0.00019	0.00580 ± 0.00031	0.00506 ± 0.00031	1.152 ± 0.308	0.01326 ± 0.02017
EAE	0.01180 ± 0.00074 ^a^	0.00597 ± 0.00035	0.00583 ± 0.00105	1.062 ± 0.235	0.11050 ± 0.02344 ^a^
EAE + EVOO	0.01088 ± 0.00057 ^d^	0.00804 ± 0.00112 ^d^	0.00283 ± 0.00075 ^d^	3.069 ± 1.000 ^d^	0.03539 ± 0.02955 ^d^
EAE + HT	0.00377 ± 0.00025 ^d,g^	0.00166 ± 0.00005 ^d,g^	0.00211 ± 0.00029 ^d^	0.802 ± 0.386 ^g^	0.00616 ± 0.00126 ^d,g^
EAE + OA	0.02201 ± 0.00131 ^d,g^	0.00609 ± 0.00215 ^g^	0.01592 ± 0.00121 ^d,g^	0.391 ± 0.552 ^d,g^	0.01283 ± 0.00190 ^d,g^
	**Kidney**				
	tG (nmol/mg protein)	GSH (nmol/mg protein)	GSSG (nmol/mg protein)	GSH/GSSG	GPx (nmol/mg protein)
Control	0.01827 ± 0.00015	0.00618 ± 0.00089	0.01209 ± 0.00095	0.518 ± 0.240	0.04728 ± 0.00149
EAE	0.01170 ± 0.00047	0.00473 ± 0.00128 ^a^	0.00698 ± 0.00152 ^a^	0.737 ± 0.137	0.04170 ± 0.01636
EAE + EVOO	0.01135 ± 0.00061	0.00866 ± 0.00057 ^d^	0.00270 ± 0.00097 ^d^	3.000 ± 1.000 ^d^	0.05123 ± 0.02927
EAE + HT	0.00305 ± 0.00059	0.00106 ± 0.00032 ^d,g^	0.00199 ± 0.00033 ^d^	0.533 ± 0.230 ^g^	0.05620 ± 0.02231
EAE + OA	0.01970 ± 0.00161	0.00749 ± 0.00073 ^d,g^	0.01221 ± 0.00136 ^d,g^	0.619 ± 0.087 ^g^	0.05642 ± 0.02262
	**Liver**				
	tG (nmol/mg protein)	GSH (nmol/mg protein)	GSSG (nmol/mg protein)	GSH/GSSG	GPx (nmol/mg protein)
Control	0.01078 ± 0.00025	0.00773 ± 0.00128	0.00305 ± 0.00123	3.000 ± 2.000	0.09980 ± 0.00756
EAE	0.03050 ± 0.04167	0.00470 ± 0.00039	0.02580 ± 0.04174	0.868 ± 0.343	0.03595 ± 0.02465
EAE + EVOO	0.01121 ± 0.00031	0.03236 ± 0.04630	0.02116 ± 0.04628	5.000 ± 7.081 ^d^	0.08238 ± 0.01286
EAE + HT	0.00473 ± 0.00048 ^d^	0.00154 ± 0.00019 ^d,g^	0.00319 ± 0.00052	0.494 ± 0.240 ^g^	0.19775 ± 0.05116
EAE + OA	0.02200 ± 0.00049	0.00782 ± 0.00034 ^g^	0.01418 ± 0.00066	0.553 ± 0.047 ^g^	0.26600 ± 0.07108

Total glutathione (tG; nmol/mg protein), reduced glutathione (GSH; nmol/mg protein), and oxidized glutathione (GSSG; nmol/mg protein); glutathione peroxidase (GPx; nmol/mg protein) and GSH/GSSG ratio, in the following study groups: control group (not manipulated); EAE group (experimental autoimmune encephalomyelitis induced by myelin oligodendrocyte glycoprotein (MOG)); EAE + extra-virgin olive oil (EVOO); EAE + hydroxytirosol group (HT); and EAE + oleic acid group (OA). ^a^ *p* < 0.001 EAE vs. control; ^d^ *p* < 0.001 vs. EAE; ^g^ *p* < 0.001 vs. EAE + EVOO.

**Table 2:** Oxidative stress products in the heart, kidney, and liver.

Oxidative Stress Products		
**Heart**		
	LPO (nmol/mg protein)	CP (nmol/g protein)
Control	0.11158 ± 0.02087	0.03420 ± 0.00388
EAE	0.41875 ± 0.00000 ^a^	0.41875 ± 0.02344 ^a^
EAE + EVOO	0.09625 ± 0.02918 ^d^	0.02249 ± 0.00986 ^d^
EAE + HT	0.00769 ± 0.00065 ^d^	0.00616 ± 0.00126 ^d,g^
EAE + OA	0.02064 ± 0.00496 ^d,g^	0.01283 ± 0.00190 ^d^
**Kidney**		
	LPO (nmol/mg protein)	CP (nmol/g protein)
Control	0.19280 ± 0.03422	0.00743 ± 0.00112
EAE	0.45750 ± 0.01063 ^a^	0.08493 ± 0.02702^a^
EAE + EVOO	0.08875 ± 0.01001 ^d^	0.02920 ± 0.01707 ^d^
EAE + HT	0.00683 ± 0.00111 ^d,g^	0.00326 ± 0.00039 ^d^
EAE + OA	0.01867 ± 0.00159 ^d,g^	0.01989 ± 0.00181 ^d^
**Liver**		
	LPO (nmol/mg protein)	CP (nmol/g protein)
Control	0.11140 ± 0.02013	0.01076 ± 0.00220
EAE	0.40250 ± 0.07214 ^a^	0.09568 ± 0.02642 ^a^
EAE + EVOO	0.10663 ± 0.00616 ^f^	0.03815 ± 0.01375 ^d^
EAE + HT	0.00826 ± 0.00189 ^d,g^	0.00660 ± 0.00083 ^d,h^
EAE + OA	0.02768 ± 0.00533 ^d,g^	0.03332 ± 0.00207 ^d^

Lipid peroxidation products (LPO; nmol/mg protein) and carbonylated proteins (CP; nmol/g protein) in the following study groups: control group (not manipulated); EAE group (experimental autoimmune encephalomyelitis induced by myelin oligodendrocyte glycoprotein (MOG)); EAE + extra-virgin olive oil (EVOO); EAE + hydroxytirosol group (HT); and EAE + oleic acid group (OA). ^a^ *p* < 0.001 EAE vs. control; ^d^ *p* < 0.001 vs. EAE; ^f^ *p* < 0.05 vs. EAE; ^g^ *p* < 0.001 vs. EAE + EVOO; ^h^ *p* < 0.01 vs. EAE + EVOO.

**Table 3:** Pearson’s correlation between lipopolysaccharide (LPS) of the external gram-negative bacteria wall and the lipopolysaccharide binding protein (LBP) with lipid peroxidation products (LPO) and carbonylated proteins (CP) in the small and large intestine. *r*-value and (*p*-value).

Pearson’s Correlation		
**Small Intestine**		
	LPO	CP
LPS	0.636 (0.001)	0.542 (0.008)
LBP	0.816 (0.000)	0.777 (0.000)
**Large Intestine**		
	LPO	CP
LPS	0.759 (0.000)	0.581 (0.004)
LBP	0.703 (0.000)	0.747 (0.000)
